# Brazilian organic propolis for prevention and treatment of radiation-related oral acute toxicities in head and neck cancer patients: A double-blind randomized clinical trial

**DOI:** 10.3389/fphar.2022.973255

**Published:** 2022-10-07

**Authors:** Patrícia Maria Fernandes, Pedro Luiz Rosalen, Diego Tetzner Fernandes, Emmanuel Dias-Neto, Severino Matias Alencar, Bruno Bueno-Silva, Fábio de Abreu Alves, Márcio Ajudarte Lopes

**Affiliations:** ^1^ Oral Diagnosis Department, Piracicaba Dental School, University of Campinas (UNICAMP), Piracicaba, São Paulo, Brazil; ^2^ Department of Bioscience, Piracicaba Dental School, University of Campinas (UNICAMP), Piracicaba, São Paulo, Brazil; ^3^ Biological Sciences Graduate Program, Federal University of Alfenas, Alfenas, Minas Gerais, Brazil; ^4^ Laboratory of Medical Genomics, Research International Center (CIPE), A.C. Camargo Cancer Center, São Paulo, São Paulo, Brazil; ^5^ Department of Agri-food Industry, Food and Nutrition, “Luiz de Queiroz” College of Agriculture, University of São Paulo, Piracicaba, São Paulo, Brazil; ^6^ Dental Research Division, Guarulhos University, Guarulhos, São Paulo, Brazil; ^7^ Stomatology Department, A.C. Camargo Cancer Center, São Paulo, São Paulo, Brazil

**Keywords:** oral mucositis (OM), dysphagia, dysgeusia, oral candidiasis, cytokines, head and neck cancer

## Abstract

**Background:** Oral mucositis (OM) is one of the most important acute toxicities from radiotherapy (RT) in head and neck cancer patients and can impair oncologic treatment. Dysphagia, dysgeusia, pain, and oral candidiasis are other common toxicities. Brazilian Organic Propolis (BOP) is a recently described propolis variant and BOP types 4 and 6 have shown important antioxidant, anti-inflammatory, and antifungal properties.

**Purpose:** To investigate the use of BOP as a preventive and/or complementary therapeutic option for radiotherapy-induced oral mucositis, dysphagia, dysgeusia, pain, and oral candidiasis. Additionally, proinflammatory cytokines were assessed to investigate their anti-inflammatory role.

**Methods:** Sixty patients were included in this randomized, double-blind, controlled clinical trial. Patients were randomized to receive either aqueous suspension of a BOP or placebo throughout RT. Also, all patients underwent low-level laser therapy as routine oral care. OM, dysphagia, and dysgeusia were assessed weekly according to WHO and NCI scales. Pain-related to OM was assessed according to a Visual Analog Scale and the presence or absence of oral candidiasis was checked by intraoral examination. Protein levels of TNF-α and IL-1β from oral mucosa were assessed by ELISA.

**Results:** Patients in the propolis group had a lower mean score of OM, dysphagia, dysgeusia, and most patients reported moderate pain. Fewer patients developed oral candidiasis in the propolis group, and the number of episodes was lower among patients that used BOP (*p* < 0.05). In addition, the BOP group presented significantly lower levels of IL-1β since the beginning of treatment when compared with placebo patients (*p* < 0.05) and a lower level of TNF-α at the end of treatment (*p* < 0.001).

**Conclusion:** Topic use of BOP reduced TNF-α and IL-1β levels, oral candidiasis episodes, and seems to be a useful complementary option for the prevention and treatment of the main acute oral toxicities of RT.

**Clinical Trial Registration:** http://www.ensaiosclinicos.gov.br/rg/RBR-9f8c78/, identifier RBR-9f8c78

## Introduction

Multimodal treatment is part of the head and neck cancer (HNC) patients setting. In this sense, radiotherapy (RT) plays a central role either in early or advanced-stages tumors. Usually, oral and oropharyngeal cancer patients undergo high-dose of radiation delivered daily for approximately 7 weeks. However, RT induces a variety of dose-dependent acute and chronic toxicities that correlate with each other and impairs treatment outcomes, decreasing the quality of life of patients (Mazeron et al., 2009; Shah and Gil, 2009; Fridman et al., 2018).

In the set of acute toxicities, oral mucositis (OM) represents the most significant side-effect in patients who underwent RT in the head and neck region (Carvalho et al., 2011; Villa and Sonis, 2020). This condition is characterized by the onset of erythema and soreness in oral mucosa that, progressively, evolve with ulcerative lesions (Sonis, 2009; Villa and Sonis, 2020). Biological mechanisms involved in OM include activation of transcription factors such as NF-kB, releasing pro-inflammatory cytokines such as tumor necrosis factor-alpha (TNF-α) and interleukin 1-beta (IL-1β). This molecular pathway produces a positive feedback chain that amplifies tissue damage (Sonis, 2009; Normando et al., 2017). Pain is a recurrent complaint, but OM also leads to changes in food choices, and decrease oral hygiene. Until now, there are no pharmacological or non-pharmacological interventions capable of completely avoiding the onset of this condition (Sonis, 2009; Villa and Sonis, 2020). Besides OM, patients also suffer from swallowing disturbances (dysphagia), taste and smell alterations (dysgeusia), dry mouth (xerostomia), and are assaulted by opportunistic infections, being oral candidiasis the most common in the oral cavity. Altogether, high-grade of these acute toxicities imply in treatment interruptions, profound loss of quality of life, and a decreased overall performance during treatment (Sonis, 2009; Porter et al., 2010; Mercadante et al., 2015; Moroney et al., 2017; Frowen et al., 2020; Villa and Sonis, 2020).

For centuries, natural products have been used for a wide variety of diseases. A recent review showed that within the non-synthetic molecules approved by the U.S. Food and Drug Administration (FDA) in the cancer field in general, 53% were from natural products or derived compounds (Newman and Cragg, 2020). In this context, bee-derived products have been targeted as potential agents for managing radiation and/or chemotherapy-related toxicities (Khanal et al., 2010; Abdulrhman et al., 2012; Bardy et al., 2012; Hawley et al., 2014; Cho et al., 2015; Samdariya et al., 2015; Marucci et al., 2017; Münstedt and Männle, 2019).

Among bee-derived products, propolis is a resin collected by bees from the buds and exudates of plants in the beehive area and has stood out because of bioactive compounds (Bueno-Silva et al., 2017). Noteworthy, propolis is considered as a functional food and generally recognized as safe (GRAS) product by FDA (El-Guendouz et al., 2018; Nani et al., 2020).

Brazilian Organic Propolis (BOP) (Kiwa BCSÖko-Garantie GmbH, 2016) is a new kind of propolis recently described, characterized by its mild flavor and absence of heavy metals and pesticides. Produced under organic conditions in conservation areas, BOP has shown interesting bioactive properties. Seven different chemical profiles were identified (Tiveron et al., 2016; Nani et al., 2020). Among them, BOP type 4 presented important antioxidant activity due to its high content of Artepillin C. BOP type 6 showed a remarkable anti-inflammatory activity through decreasing NF-kB activation and TNF-α release. Once the pathogenesis of OM is closely related to inflammatory pathways and reactive oxygen species production, both types of BOP became a target of investigation. In addition, BOP type 6 presented a substantial effect against *Candida albicans* and non-*albicans* species in a previous study (Nani et al., 2020). However, despite the excellent BOP pre-clinical data, none of the controlled clinical trials are evaluating it in the literature.

Considering the previous promising findings on BOP pharmacological properties and the challenge of management of acute toxicities of radiation therapy in the head and neck region, this prospective study aimed to investigate the use of propolis as a preventive and/or complementary therapeutic option for OM as well as in dysgeusia, dysphagia, pain, and oral candidiasis. Additionally, protein levels of the inflammatory cytokines TNF-α and IL-1β were assessed to investigate the potential anti-inflammatory role of BOP in a subclinical way.

## Patients and methods

### Study design

This study consisted of a randomized, double-blind, and controlled clinical trial. It has been approved by the Ethics Committee of Piracicaba Dental School, University of Campinas, Piracicaba, Brazil (CAAE: 61163616.2.0000.5418) and the Ethics Committee of A.C. Camargo Cancer Center, São Paulo, Brazil (CAAE: 61163616.2.3001.5432). This study complied with the Declaration of Helsinki and was registered at the Brazilian Clinical Trials Registry—ReBEC (RBR-9f8c78). Also, this study followed the guideline for reporting clinical trial studies per the Consolidated Standards of Reporting Trials (CONSORT 2010) statement (Schulz et al., 2010).

### Sample size estimation and randomization

Sample size was determined considering a confidence level of 95% (*α* = 0.05), power of 80% (1 <β> =0.80) and an expected loss ratio of 15% (Carvalho et al., 2011). Hence, an ideal number of 30 patients per group was established.

The study participants were randomly allocated into 2 groups. A randomization list in a non-stratified manner was generated using *R software*—Version 3.4.2; Vienna, Austria (R Core Team, 2013) to determine who would receive either BOP or placebo.

### Patients

To be eligible for the study, participants had to be aged 18 years or older, diagnosed with oral cavity or oropharynx cancer and be undergoing RT three-dimensional conformal radiotherapy (3DRT) or intensity-modulated radiation therapy (IMRT) with a dose of at least 40 Gy either adjuvant to surgery, exclusively or associated with chemotherapy.

Patients with a known history of allergy to propolis itself or any of its compounds, as well as patients who have been submitted to previous RT in the head and neck region were excluded.

**FIGURE 1:**
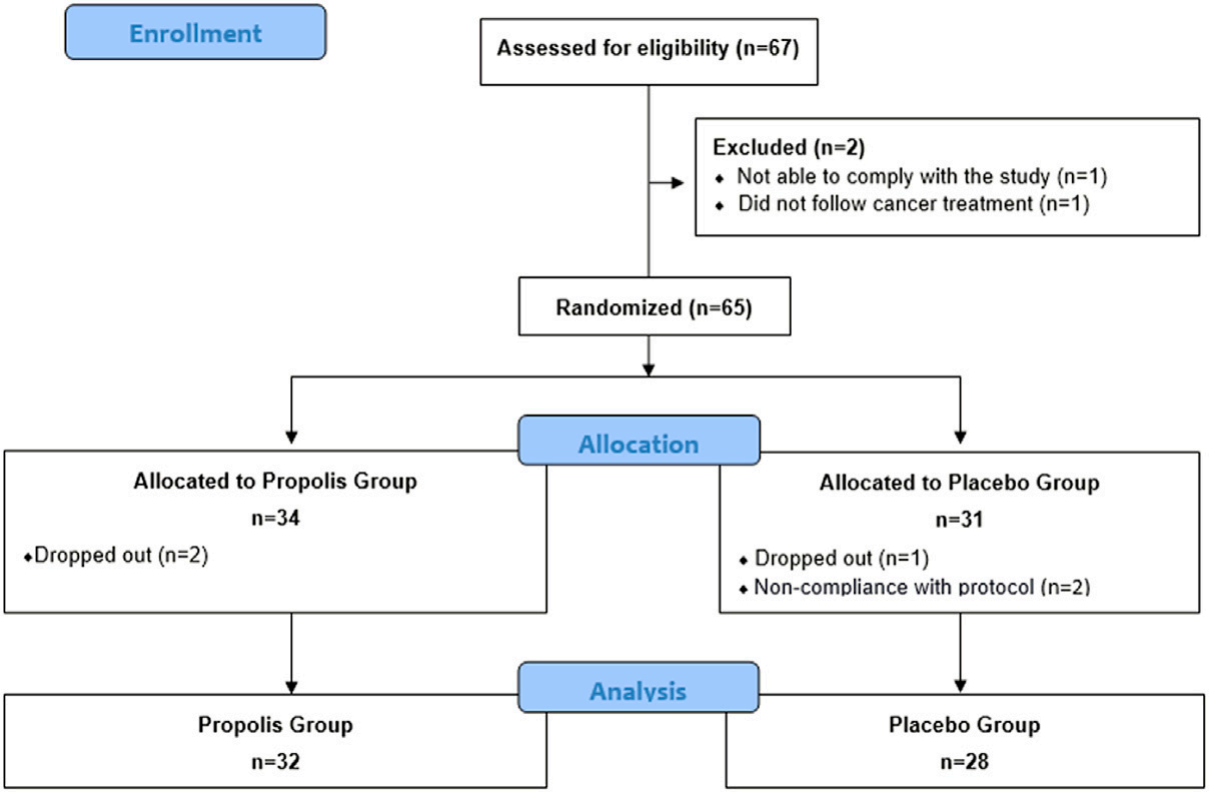
Consort 2010 flow diagram showing the recruitment of participants.

The study took place at A.C. Camargo Cancer Center, São Paulo, Brazil. Between March 2018 and February 2020, patients were assessed for eligibility and all patients included signed an informed consent form. A CONSORT 2010 flow diagram (Figure 1) details the recruitment of participants.

### Intervention

The collection of BOP types 4 and 6 were carried out between February 2015 and December 2016 in the respective georeferenced sites: city of Palmital (24°53′S, 52°12′W) and Campo Largo (25°27′S, 49°31′W), Paraná state, Brazil.

The production of hydroethanolic extracts (80°GL) were performed as described by Tiveron et al. (2016) and Nani et al. (2020). The extracts of BOP types 4 and 6 were compared to the previous profiles described by Tiveron and colleagues (2016) and both samples presented a similar chromatographic profile in TLC and HPLC assays (image available in supplementary material section).

For the chemical profile characterization and identification of the main compounds present in BOP type 4 and 6, the high definition accurate-mass liquid chromatography electrospray ionization quadrupole time-of-flight mass spectrometry (LC–ESI-QTOF-MS) experiment was performed. A HPLC equipment (Shimadzu Co., Kyoto, Japan) coupled to a high-resolution quadrupole time-of-flight spectrometer (MAXIS 3G, Bruker Daltonics, Bremen, Germany), equipped with an electrospray ionization (ESI) source operating in negative mode was used. Chromatographic separation was performed on a Phenomenex Luna C18 column (4.6 × 250 mm × 5 μm). The mobile phase consisted of a mixture of two solvents: 1) 0.5% acetic acid and 99.5% water; and 2) methanol 100%. The flow rate of the mobile phase was 0.8 ml/min. The gradient was started with 30% of solvent B; increasing to 60% in 45 min, 75% in 85 min, 90% in 90 min, resuming to 30% in 105 min and finishing the run in 105 min. Before the analysis, an external calibration was performed to determine the accuracy of the masses measured. Data analysis was performed using MAXIS 3G software (Bruker Daltonics, version 4.3), and the identification of the compounds was made by comparing the exact masses, MS/MS mass spectra and molecular formulas to the database available in the literature (Nani et al., 2020).

BOP genetic heritage was registered on the SISGEN platform (number A0277F6) following the Brazilian legislation SECEX/CGEN. Also, BOP was under the rules of international certification of organic production and handling operations (National Organic Program—NOP) from the United States Department of Agriculture and CEE (European Union).

An aqueous suspension containing 0.8% (w/v) of the BOP mix [50% (w/w) of BOP type 4 and 50% (w/w) of BOP type 6] was elaborated by Breyer and Cia Ltda (União da Vitória, Paraná State, Brazil) under in-house custom process and bottled in 30 ml amber spray bottle. Ethanol residual was measured lower than 0.015 ml per 100 ml of product.

The same company provided identical amber spray bottles containing a placebo which consisted of distilled water.

### Patient evaluation and outcome assessment

Before the beginning of RT, each patient received an anonymized amber spray bottle and both patients and professionals were unaware of the bottle content. The patient was instructed to apply the product topically upon the oral mucosa at least 6 times daily, every day, including weekends since day 1 of RT. Patients were constantly encouraged to keep using the product until the last day of RT.

All patients scheduled for RT underwent oral care protocol, including oral examination, dental treatment when needed, instructions about oral care during RT, and prescription mouthwashes among others. During RT, all patients underwent preventive and, if necessary, curative low-level laser therapy (LLLT) as routine care in the Department of Stomatology of A.C. Camargo Cancer

Clinical evaluation was performed weekly through intraoral examination by a single dentist in a blinded fashion. OM was assessed according to the World Health Organization Criteria (WHO-1979) and the National Cancer Institute Common Terminology Criteria for Adverse Events (NCI CTCAE, Version 4.0, 2010) scales.

Dysgeusia, dysphagia, oral candidiasis, and pain related to OM were also evaluated. Dysgeusia and dysphagia were assessed weekly according to the NCI (Version 2.0, 1999) scales. In every appointment, patients rated their pain according to a visual analog scale (VAS), from 0 (absence of pain) to 10 (the worse pain sensation). Scores between 4 and 7 were considered moderate pain (Carvalho et al., 2011).

### TNF-α and IL-1β measurement

Protein levels of TNF-α and IL-1β of 48 patients (23 from the propolis group and 25 from placebo group) were assessed in three time-points: before treatment, between 10 and 20 Gy and at the end of RT (after 48 Gy). Samples were collected using a swab, which was gently rubbed in whole oral mucosa and immediately frozen and stored at −80°C until analysis. For samples extraction, 300 µl of phosphate buffer saline was added to the collected swab, and vortex for 1 min. Finally, samples were then centrifuged at 10,000 rpm for 10 min at 4°C. The supernatants were collected and stored at −20°C until further analysis.

All samples were quantified by Enzyme-linked immunosorbent assay (ELISA). The kits were purchased from Becton-Dickinson (San Diego, CA, United States) and the assays were performed according to the manufacturer’s recommendations.

### Statistical analysis

Research data were collected and managed using the Research Electronic Data Capture software (*REDCap*) hosted at the A.C. Camargo Cancer Center (Harris et al., 2009; Harris et al., 2019). Clinical data and demographic information were obtained from medical records and organized as well as managed in a like manner.

Data analysis was performed using SPSS, version 23.0 (IBM Corp., Armonk, NY, United States), R software, Version 3.4.2 (Vienna, Austria), and GraphPad Prism 9.3. Clinicopathological features were established through absolute and relative frequencies. To compare clinicopathological features between groups Chi-square and Fisher tests were used. Non-parametric variables were analyzed through the Mann-Whitney test. Survival curves were performed using the Kaplan-Meier method, and a Long-rank test was carried out to evaluate the onset of OM during RT treatment. When comparing OM, dysphagia, dysgeusia, and pain between the two groups, the areas under the curve of the respective scores of the 6 first weeks of RT were calculated and compared through the Student´s *t*-test. The cytokine data were analyzed using two-way ANOVA, followed by Tukey’s test. The significance level was fixed at 5% for all statistical tests.

## Results

### Clinicopathological findings

A total of 65 patients were enrolled in the trial. Five patients were excluded during RT (2 in propolis group and 3 in placebo group) due to the absence of RT or daily appointments to the stomatology department (*n* = 2) or by their own choice (*n* = 3). Thereby, a total of 60 patients completed their participation in the study (Figure 1). Clinical and pathological data are detailed in Table 1.

**TABLE 1:** Demographic and clinicopathological features of patients in study.

Clinical features	Propolis group (*n* = 32) n patients (%)	Placebo group (*n* = 28) n patients (%)	Total (*n* = 60) n patients (%)	*p* value
Age (years)				
Mean (SD)	58 (9.7)	59.5 (10.2)	58.7 (9.9)	0.56†
Range	37–80	42–86	37.6–86.4	
Sex				
Male	22 (68.8)	23 (82.1)	45 (75)	0.37**
Female	10 (31.3)	5 (17.9)	15 (25)	
Smoking status				
Never	11 (34.4)	4 (14.3)	15 (25)	0.15**
Current	10 (31.3)	14 (50)	24 (40)	
Former	11 (34.4)	10 (35.7)	21 (35)	
Alcohol consumption history				
No	7 (22.6)	4 (14.8)	11 (19)	0.67**
Yes	24 (77.4)	23 (85.2)	47 (81)	
Histopathological diagnosis				
Squamous cell carcinoma	31 (96.9)	26 (92.9)	57 (95)	0.44*
Verrucous carcinoma	0	1 (3.6)	1 (1.7)	
Cystic Adenoid carcinoma	0	1 (3.6)	1 (1.7)	
Mucoepidermoid carcinoma	1 (3.1)	0	1 (1.7)	
Macrolocation				
Mouth	17 (53.1)	12 (42.9)	29 (48.3)	0.59**
Oropharynx	15 (46.9)	16 (57.1)	31 (51.7)	
Specific location				
Mouth				
Lateral/ventral surface of the tongue	9 (28.1)	2 (7.1)	11 (18.3)	0.14*
Mouth floor	2 (6.3)	6 (21.4)	8 (13.3)	
Hard palate	3 (9.4)	1 (3.6)	4 (6.7)	
Gingiva/alveolar mucosa	2 (6.3)	1 (3.6)	3 (5)	
Buccal mucosa	1 (3.1)	0	1 (1.7)	
Retromolar region	0	2 (7.1)	2 (3.3)	
Oropharynx				
Amygdala	4 (12.5)	8 (28.6)	12 (20)	
Base of the tongue	6 (18.8)	5 (17.9)	11 (18.3)	
Soft palate	3 (9.4)	1 (3.6)	4 (6.7)	
Pharingeal wall	2 (6.3)	2 (7.1)	4 (6.7)	
Stage				
I	2 (6.7)	0	2 (3.5)	0.7*
II	3 (10)	3 (11.1)	6 (10.5)	
III	10 (33.3)	8 (29.6)	18 (31.6)	
IV	15 (50)	16 (59.3)	31 (54.4)	
Treatment				
Sur+RT	14 (43.8)	7 (25)	21 (35)	0.57*
Sur+CRT	7 (21.9)	7 (25)	14 (23.3)	
CRT	6 (18.8)	7 (25)	13 (21.7)	
CT+CRT	2 (6.3)	5 (17.9)	7 (11.7)	
CT+Sur+CRT	2 (6.3)	1 (3.6)	3 (5)	
RT	1 (3.1)	1 (3.6)	2 (3.3)	
RT modality				
3D	8 (25)	14 (51.9)	22 (37.3)	0.06**
IMRT	24 (75)	13 (48.1)	37 (62.7)	
Dose RT (Gy)				
≤60	19 (59.4)	12 (42.9)	31 (51.7)	0.3**
>60	13 (40.6)	16 (57.1)	29 (48.3)	
Range	50–70	50–80	50–80	

^†^, t test; *, Fisher; **, Pearson; Sur, Surgery; RT, radiotherapy; CRT, chemoradiotherapy; CT, induction chemotherapy.

### Evaluation of oral mucositis

**FIGURE 2:**
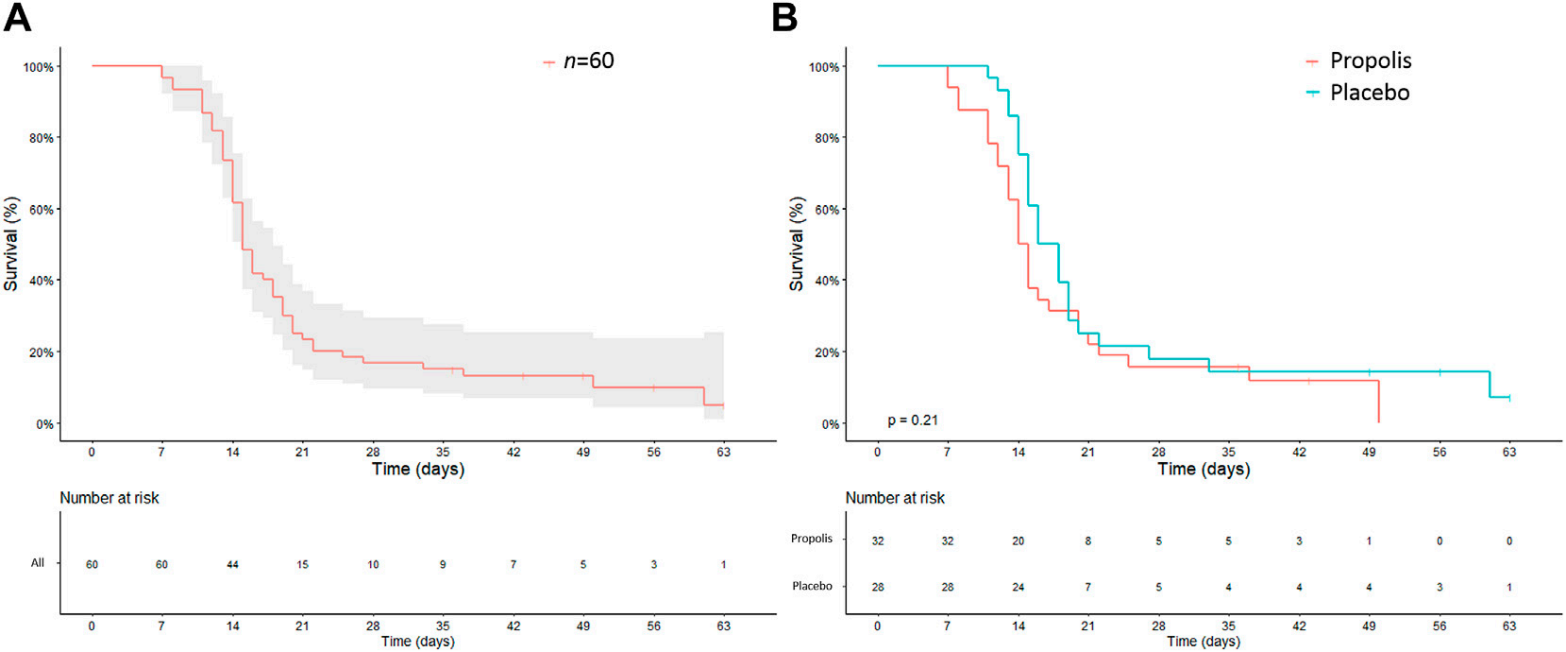
Kaplan-Meier curves for oral mucositis grade II outcome among all patients **(A)** and who receive either Brazilian Organic Propolis or placebo **(B)**.

Considering the whole cohort (*n* = 60), 59 patients (98.4%) developed some degree of OM during treatment. Figure 2A shows an overview of the onset of OM grade II. When the groups were compared separately, Kaplan-Meier analysis showed no significant reduction in the onset of OM grade II in propolis group when compared to placebo group (*p* = 0.21), as highlighted in Figure 2B.

**FIGURE 3:**
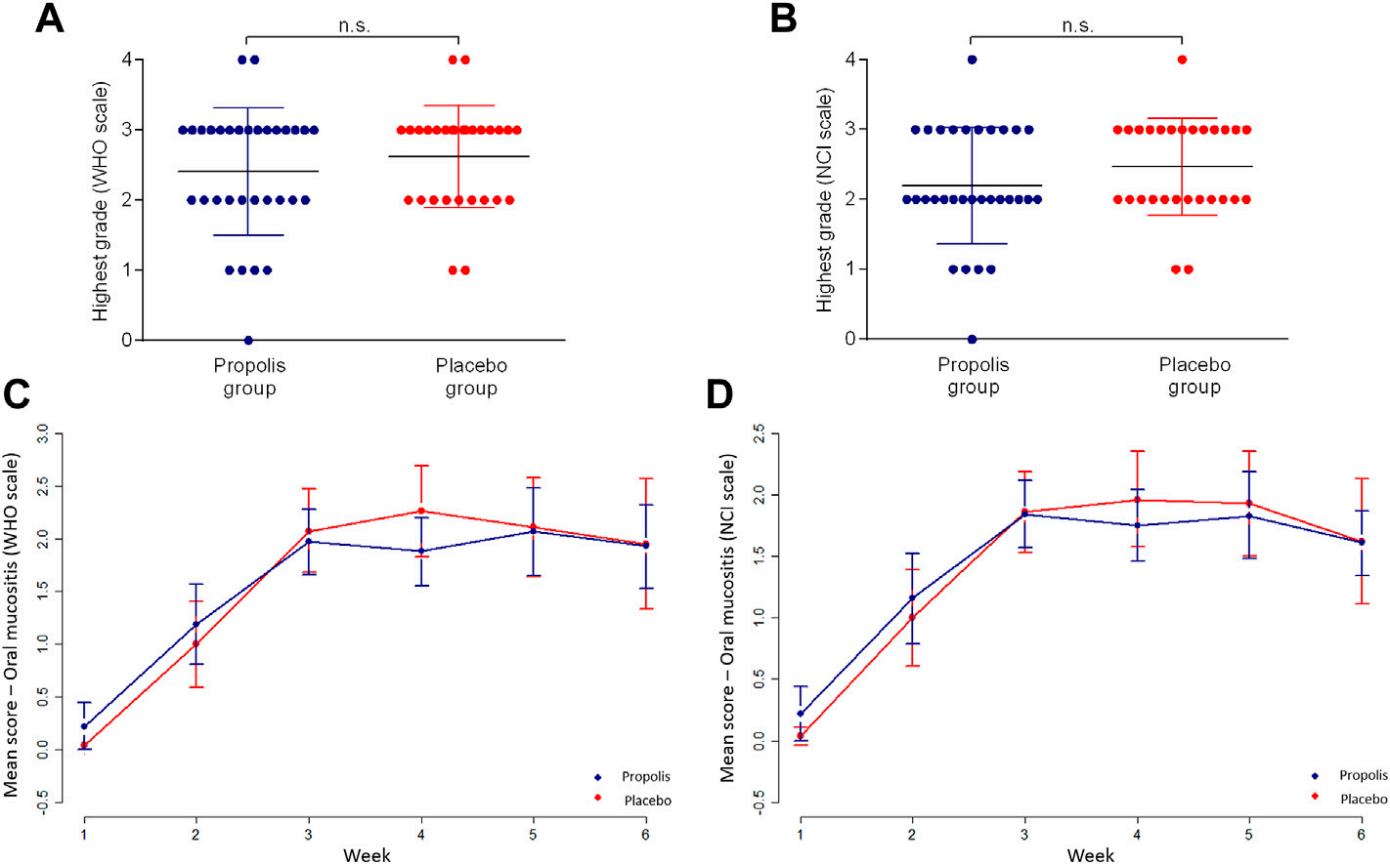
The highest grade of oral mucositis that each patient developed according to WHO **(A)** and NCI **(B)** scales. Weekly mean score of oral mucositis according to WHO **(C)** and NCI **(D)** scales.

Considering WHO scale, 15 (46.8%) patients developed mild OM whereas 17 (53.1%) reached severe OM among patients who used BOP. Among those who used placebo, 10 (35.5%) patients developed mild OM whereas 18 (64.2%) reached severe OM. The same outcome was observed considering the NCI scale for grades 0 and I. While the majority of patients in propolis group remained in OM grade II (16 patients—50%), most patients in placebo group reached OM grade III (13 patients—46.4%). One patient reached OM grade IV in propolis group (3.1%) as well as in placebo group (3.5%) (see Figures 3A,B).

In the weekly evaluation, for both groups, the highest mean score occurred after the third week of RT. Propolis group presented a lower mean of OM grade than placebo group especially in the fourth week, exactly when patients in placebo group achieved the peak of OM, according to WHO scale and NCI scale. However, no significant differences between the two groups were evident (Figures 3C,D).

### Dysphagia

All patients developed some inability to swallow during RT treatment. In propolis group, 23 patients (71.8%) reported the use of diets predominantly liquid or pasty (grade II) and 5 patients (15.6%) needed to be tube fed (grade III). In placebo group, 1 patient (3.5%) reported moderate dysphagia but with normal diet (grade I), 17 patients (60.7%) reported liquid and pasty diet, 4 patients (14.2%) needed to be tube fed and 1 patient (3.5%) needed a gastrostomy (grade IV) (*p* = 0.92). Finally, 9 patients (15%) [4 patients in propolis group (12.5%), and 5 (17.8%) in placebo group] were unavailable for analysis due to impossibility to oral intake before the start of RT.

**FIGURE 4:**
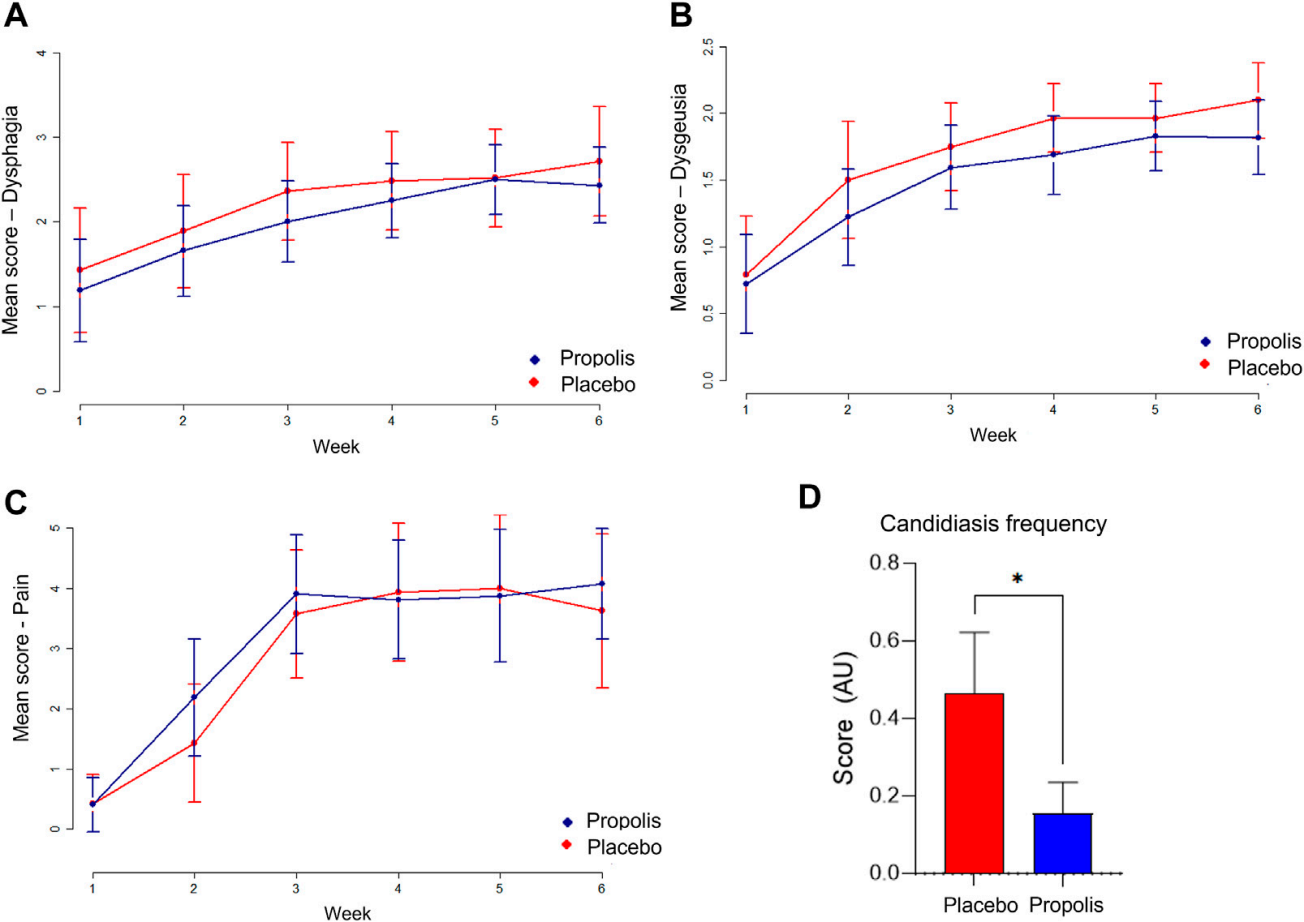
Weekly mean score of dysphagia **(A)**, dysgeusia **(B)**, pain **(C)** and bar diagram showing candidiasis frequency **(D)**.

Participants in propolis group had a mean score lower than placebo group from the first to the fourth week as well as on the sixth (Figure 4A). However, no significant differences were found between groups.

### Dysgeusia

Only 3 patients (5%: 2/6.2% in propolis group and 1/3.5% in placebo group) did not report taste function alteration during treatment. In propolis group, 4 patients (12.5%) reported mild alteration in taste (grade I) and 3 patients in placebo group (10.7%). Twenty-two patients (68.7%) reported strong taste alteration (grade II) in propolis group and 19 patients in placebo group (67.8%) (*p* = 1.0). Likewise, the same subjects above excluded from *dysphagia* analysis were excluded from this analysis as well.

Weekly evaluation showed a continuous evolution of dysgeusia scenario for both groups, with the highest scores being reached at the end of the treatment. Although propolis group participants always presented a lower overall mean score, none of these differences were statistically significant (Figure 4B).

### Pain related to oral mucositis

For both groups, most patients experienced moderate pain (scoring from 4 to 7). The mean score was 5.36 in propolis group and 5.33 in placebo group (*p* = 0.99). In weekly evaluation, the higher pain score occurred on the third and sixth weeks for propolis group and fourth and fifth weeks for placebo group (Figure 4C). No significant reduction in pain was found in propolis group when compared to placebo group.

### Oral candidiasis

Twelve patients (20%) developed oral candidiasis during RT (4/42%) patients were in propolis group and 8/28.5% in placebo group. In propolis group, 3 patients had 1 single episode of oral candidiasis, and 1 patient had 2 episodes during treatment. In placebo group, 4 patients had 1 single episode, 3 patients had 2 episodes and 1 patient had 3 episodes of oral candidiasis. Overall, candidiasis frequency was significantly lower (*p* < 0.05) in propolis group than placebo group (see Figure 4D).

### Brazilian organic propolis reduces inflammatory cytokines in oral mucositis

**FIGURE 5:**
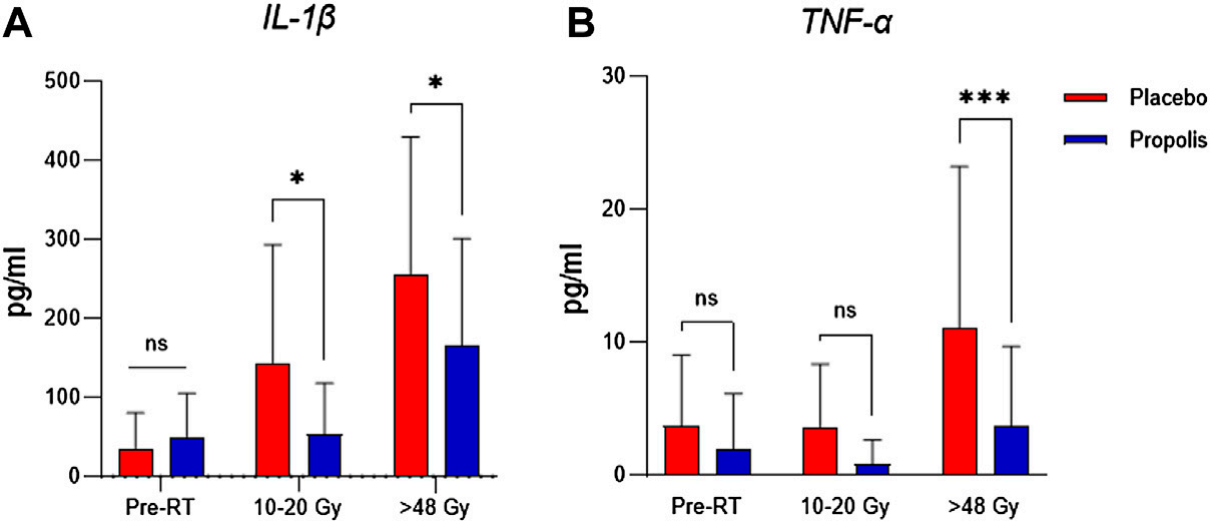
Pro-inflammatory cytokine IL1-β **(A)** is reduced in the early treatment phase whereas TNF-α **(B)** is decreased in final phase.

The analysis of protein levels of inflammatory cytokines revealed that patients who used BOP presented significant lower levels of IL-1β since the beginning of treatment when compared with those who used placebo (*p* < 0.05) (Figure 5A). TNF-α also was diminished in patients who used BOP at the end of treatment (*p* < 0.001) when radiation dose was equal or higher than 48 Gy (Figure 5B).

### Chemical profile and main compounds of Brazilian organic propolis

Ten compounds were identified in BOP used in the present study. Caffeoyltartaric acid, 3,4-dicaffeoylquinic acid, quercetin, gibberellins and artepillin C are some of them. The complete compound´s description can be seen in Table 2.

**TABLE 2:** Phytochemical profile by LC-ESI-QTOF-MS of Brazilian Organic Propolis mix.

Peak number	Compound name	Molecular formula	Exact Mass (g/mol)	[M-H]^-^	Fragments MS (*m/z*)
1	Gallic acid	C_7_H_6_O_5_	170.0216	169.0143	**125.0239**
2	Caffeic acid	C_7_H_6_O_4_	180.0424	179.0351	**135.0452**
3	Coumaric acid	C_9_H_8_O_3_	164.0474	163.0402	**147.0443**
4	Caffeoyltartaric acid	C_13_H_11_O_9_	312.0482	311.0417	**135.0300**; 149.0288; 179.0133
5	3,4-Dicaffeoylquinic acid	C_25_H_24_O_12_	516.1270	515.1159	135.0305; 161.0066; **179.0145**
6	Quercetin	C_15_H_10_O_7_	302.0430	301.0367	124.0018; **151.9938**; 227.0444
7	Gibberellin A7	C_19_H_22_O_5_	330.1467	329.1379	**255.1454**
8	Gibberellin A20	C_19_H_24_O_5_	332.1624	331.1524	257.1618; **301.1453**; 331.1512
9	Gibberellin A9	C_19_H_24_O_4_	316.16746	315.1588	**315.1582**
10	Artepillin C	C_19_H_24_O_3_	300.1725	299.1641	255.1760; **200.1198**; 145.0683

Bold values are the main fragments.

## Discussion

As demonstrated in the clinicopathological profile of our cohort, most patients were diagnosed with advanced-stage disease, which requires multimodal treatment resulting in a variety of acute and chronic toxicities (de Pauli Paglioni et al., 2020; Villa and Sonis, 2020).

Regarding OM development, our results revealed that patients who made topical use of BOP had a lower mean score in the fourth week of RT, although it was not able to avoid or delay the onset of OM grade II. Despite the lack of statistically significant differences, we highlight that after the third week of RT, the patients usually experience the worst phase of treatment. The WHO scale better reflected the difference between groups, especially in the fourth week. A probable explanation is that the WHO scale considers the measurement of lesions in addition to the patient’s ability to eat and swallow properly. This result directly reflected the dysphagia outcome and proinflammatory cytokines expression, which is further discussed in this section.

Cytokines are mediators released by immune cells at the infected or injured site. Studies have shown that IL-1β tends to increase as incremental RT dose is delivered whereas results are inconsistent regarding TNF-α expression (Normando et al., 2017). Our findings showed that, in patients using BOP, IL-1β was reduced since the first week of treatment and TNF-α decreased at the ending phase. As already demonstrated by a previous study, BOP type 6 was able to inhibit neutrophil migration, NF-κB activation and TNF-α releasing *in vivo* and *in vitro* assays (Nani et al., 2020). Such mechanisms are the most studied and placed at the core of the physiopathology of OM. Additionally, it has been proven that other types of Brazilian propolis also can inhibit MAPK phosphorylation, a pathway activated mainly in the amplification phase of OM (Franchin et al., 2018). In addition, some of compounds identified in BOP (see Table 2) may be responsible for above-mentioned activities. A previous study noticed that 3,4-dicaffeoylquinic acid reduced the release of IL-1β and TNF-α in human keratinocytes (Liu et al., 2015). Also, gibberellin A7 demonstrated anti-inflammatory activity in a previous *in vitro* study (Reihill et al., 2016). Although gibberellin A7 was not identified in BOP, other variants of gibberellins (A7, A20, and A9) was found and we hypothesized that they could have similar biological properties. Herein, we demonstrated a remarkable blockage of pro-inflammatory pathways by BOP, indicating a substantial anti-inflammatory property. Altogether these results corroborate with the lower grade of OM especially after the fourth week of treatment.

Interestingly, none of the above-cited positive results reflected in the reduction of pain. Pain is multidimensional, which encompasses biological, social, and psychological aspects, also impacting in pain perception. Peripheral nerve sensitization is ordinary in many pathological conditions, including OM, characterized by intense cellular trafficking, driving an inflammatory cytokine storm release, with subsequently neuronal firing enhancement (Vanderwall and Milligan, 2019). A previous study that assessed oral pain in breast cancer patients found that patients using propolis did not require opioids for oral pain control (Piredda et al., 2017). On the other hand, another study found that pain was borderline significant among patients who use propolis (Tomaževič and Jazbec, 2013). Noteworthy, although patients did not describe pain relief sensation in propolis group, we must consider that patients who underwent any oncological treatment possess significant traumatic life events, emotional distress, chronic daily hassle, that can overwhelm the pain perception.

There are several studies regarding the properties of bee-derived products as preventive and therapeutic approaches for cancer therapy-induced OM (Khanal et al., 2010; Abdulrhman et al., 2012; Bardy et al., 2012; Tomaževič and Jazbec, 2013; Hawley et al., 2014; Cho et al., 2015; Samdariya et al., 2015; Marucci et al., 2017; Münstedt and Mannle, 2019). However, to date, there are only 3 previous trials that specifically used propolis for OM in HNC patients, all with encouraging results (Tomaževič and Jazbec, 2013; Javadzadeh Bolouri et al., 2015; AkhavanKarbassi et al., 2016). Nevertheless, most studies included only a small number of participants and failed to address the type, botanical origin, and phytochemical composition of propolis. It is common knowledge that propolis is a heterogeneous product with composition and biological properties that depend on the environment as well as specific plant species available for the bees. It should also be noted that the botanical origin and composition of propolis could be different within the same type of propolis, produced in the same country but in different biomes. Therefore, caution must be applied to previous studies, as the methodology is not a guarantee of study-reproducibility.

For dysphagia, patients in propolis group presented an overall lower mean score when compared to patients in placebo group, although we found no statistically significant differences. The swallowing difficulty has been reported to occur in 75%–95% of HNC patients during oncological treatment. This is a complex and multifactorial condition that interfaces with other toxicities such as OM, xerostomia, pain, dysgeusia, smell changes, mucus thickness, teeth and dentures issues, as well as tissue loss due to surgery (Moroney et al., 2017; Frowen et al., 2020). Taken together, the control of all the above-mentioned issues is remarkably challenging. As reported earlier, BOP is a promising anti-inflammatory agent and, once patients were allowed to swallow it, BOP may have been helpful in reducing tissue edema, and facilitating swallowing.

Dysgeusia is a taste dysfunction, which commonly affects HNC patients. The main causes include the tumor itself, RT, chemotherapy, and xerostomia. Underestimated in most studies, dysgeusia leads to a profound decreasing in enjoyment when eating leading to a diminished caloric intake, impairing performance during treatment, and loss of quality of life of patients (Porter et al., 2010). In our trial, patients in propolis group had a lower mean score of dysgeusia in all evaluated moments. Radioprotective properties of propolis have been previously reported and our results may be a consequence of BOP radioprotection capability on taste buds present in the tongue and the soft palate (Benković et al., 2009; Yalcin et al., 2016).

In our trial, twice as many patients have developed oral candidiasis in the placebo group compared to the propolis group. Within these patients, half of them developed more than 1 episode during treatment. Caffeoyltartaric acid and quercetin are some of the compounds found in BOP type 6 and, probably, these molecules are responsible for the antifungal effect (Nani et al., 2020). Because BOP is produced in a preserved environment, bees collect the resin from plants that facing fungal infections and brings key compounds to propolis composition. Also, these compounds probably synergize to provide the biological effect (El-Guendouz et al., 2018). A previous study showed that BOP type 6 was as effective as Amphotericin B against *Candida* spp. Biofilms by reducing adherence to human keratinocytes (Nani et al., 2020). The increase of permeability of membrane through ergosterol present in fungal cells and disruption of the microorganism cell wall are some of the mechanisms of action already described for natural products against *Candida* ssp. (Freires et al., 2016; Pereira et al., 2016). Previous trials demonstrated similar results of miconazole (clinical cure rates of 70%) when evaluating other kinds of propolis against candidiasis (Pina et al., 2017). Our trial evaluated only the preventive potential of BOP and patients who were diagnosed with oral candidiasis received the standard therapeutic approach.

Limitations of our study include the self-administration approach used in this study, which may be a source of potential bias. Additionally, no patient was deprived of standard care advocated in A.C. Camargo Cancer Center, including daily application of LLLT for OM. Thus, the isolated effects of propolis on OM were not possible to be vetted. Nevertheless, combined strategies aimed to avoid diminishing OM or any other cancer therapy side-effect may be performed and can enhance clinical outcomes.

Several questions remain to be answered. Why BOP was not as effective in human treatment as in preclinical studies is one of them. The heterogeneous nature of natural products, bioactive concentrations, dosage, and pharmacological formulation certainly play an important role. Other factors include the huge potential damage that RT and chemotherapy can cause to normal tissues. However, despite all these limitations, the current study offers valuable insights into the potential role of BOP in managing oral acute toxicities of RT in HNC patients. In addition, after several *in vitro* and *in vivo* pre-clinical data, the present study is a first step in developing a product to prevent and treat such RT oral toxicities. Besides, clinical trials with natural products are urgent and the literature lacks well-designed controlled clinical trials with propolis. Further research should focus on formulations that allow delivery of higher concentration, better control of clinicians over propolis administration and knowing the potential compound(s) present in the natural agent responsible for biological effects. In addition, the interaction between propolis and oral microbiota could be an interesting target, once propolis has been proven to decrease pathogenic and opportunistic microorganisms without influence on physiological microflora (Niedzielska et al., 2016).

In conclusion, the topic use of BOP was able to decrease the mean score of OM in key moments of RT, reducing the level of some of pro-inflammatory cytokines involved in OM physiopathology. Additionally, the use of BOP decreased the mean score of dysphagia and dysgeusia and reduced the episodes of candidiasis. Therefore, it seems a helpful alternative of a natural product for the prevention and treatment of most common acute oral toxicities of RT in association or not with other approved therapeutic methods.

## Data Availability

The raw data supporting the conclusion of this article will be made available by the authors, without undue reservation.

## Supplementary material

SUPPLEMENTARY FIGURE S1 RP-HPTLC of the seven standard types of Brazilian organic propolis **(A)** and Brazilian organic propolis types 4 and 6 used in this study **(B)** developed at a UV light of 366 nm, showing in an observational and qualitative manner that Brazilian Organic Propolis used in the present study corresponded to types 4 and 6 as already characterized in the literature.
