# Supplementary figures and images for: Brazilian organic propolis for prevention and treatment of radiation-related oral acute toxicities in head and neck cancer patients: A double-blind randomized clinical trial
Source: Front Pharmacol. 2022 Oct 7;13:973255. doi: 10.3389/fphar.2022.973255 (PMC9585325; doi:10.3389/fphar.2022.973255)

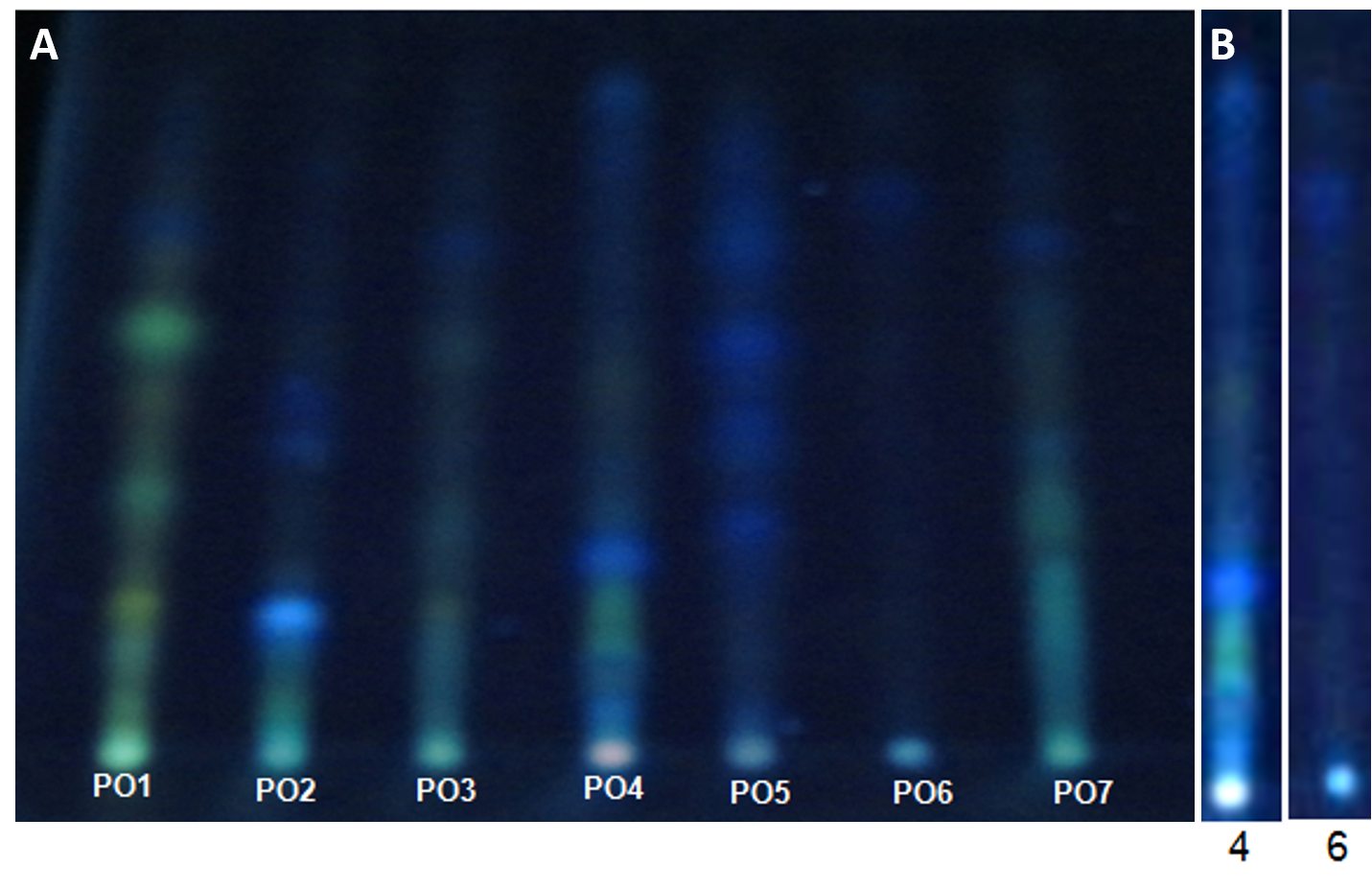

Supplement: Supplementary file 1 [file Image1.TIF]

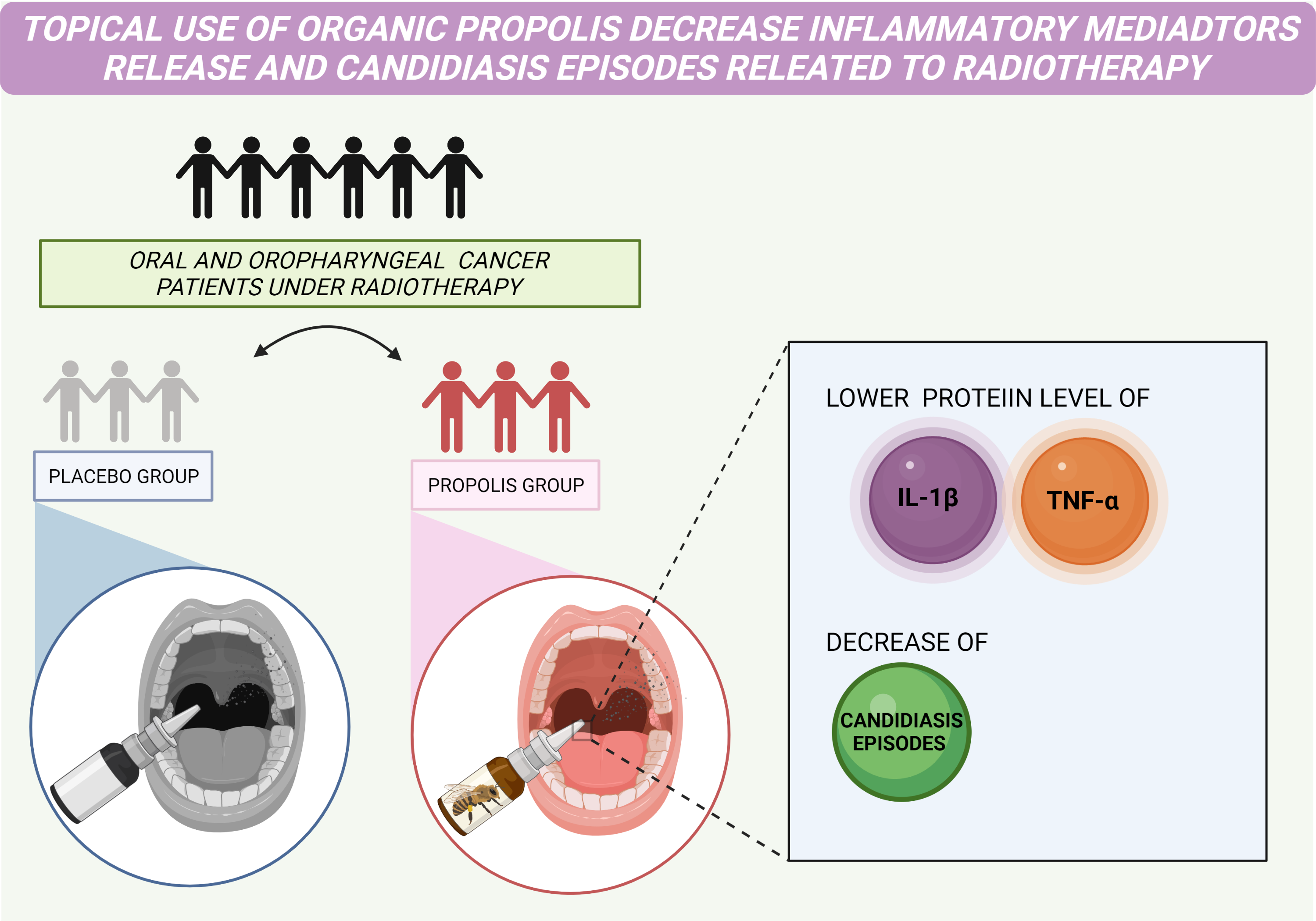

Supplement: Supplementary file 2 [file Image2.PNG]
